# Individual cells generate their own self-reinforcing contact guidance cues through local matrix fiber remodeling

**DOI:** 10.1371/journal.pone.0265403

**Published:** 2022-03-25

**Authors:** Michael Pamonag, Abigail Hinson, Elisha J. Burton, Nojan Jafari, Dominic Sales, Sarah Babcock, Rozlan Basha, Xiaofeng Hu, Kristopher E. Kubow

**Affiliations:** 1 Department of Biology, James Madison University, Harrisonburg, Virginia, United States of America; 2 Department of Chemistry & Biochemistry and Center for Materials Science, James Madison University, Harrisonburg, Virginia, United States of America; Brown University, UNITED STATES

## Abstract

Directed cell migration arises from cells following a microenvironmental gradient (e.g. of a chemokine) or polarizing feature (e.g. a linear structure). However cells not only follow, but in many cases, also generate directionality cues by modifying their microenvironment. This bi-directional relationship is seen in the alignment of extracellular matrix (ECM) fibers ahead of invading cell masses. The forces generated by many migrating cells cause fiber alignment, which in turn promotes further migration in the direction of fiber alignment via contact guidance and durotaxis. While this positive-feedback relationship has been widely described for cells invading *en masse*, single cells are also able to align ECM fibers, as well as respond to contact guidance and durotaxis cues, and should therefore exhibit the same relationship. In this study, we directly tested this hypothesis by studying the migration persistence of individual HT-1080 fibrosarcoma cells migrating in photocrosslinked collagen matrices with limited remodeling potential. Our results demonstrate that this positive-feedback relationship is indeed a fundamental aspect of cell migration in fibrillar environments. We observed that the cells’ inability to align and condense fibers resulted in a decrease in persistence relative to cells in native collagen matrices and even relative to isotropic (glass) substrates. Further experiments involving 2D collagen and electrospun polymer scaffolds suggest that substrates composed of rigid, randomly oriented fibers reduce cells’ ability to follow another directionality cue by forcing them to meander to follow the available adhesive area (i.e. fibers). Finally, our results demonstrate that the bi-directional relationship between cell remodeling and migration is not a “dimensionality” effect, but a fundamental effect of fibrous substrate structure.

## Introduction

A cell’s local environment (microenvironment) influences its migratory behavior—not only decisions about whether to migrate, but how to migrate and in which direction [1–4]. Numerous microenvironmental cues that determine the direction of cell migration have been identified including chemical, stiffness, adhesion, and magnetic gradients, cell contacts, contact guidance, and contact inhibition (see reviews: [2, 4–7]). However, it has long been known that cells are not only guided by these cues, but also play a substantial role in defining them, i.e. cells and their environment exist in a state of “dynamic reciprocity” [8] in which each affects and is affected by the other [7, 9].

A major example of dynamic reciprocity in mesenchymal-like cell migration is the interplay between cell invasion and extracellular matrix (ECM) remodeling. Cells invading from a tissue, tumor, or other localized placement into an unpopulated fibrous ECM cause widespread fiber alignment and densification (e.g. [10–13]). This matrix remodeling in turn promotes directed cell migration (e.g. [12, 13]) by restricting adhesions and protrusions [14–18] and increasing local stiffness [19, 20] and adhesive sites [21] in the direction of increased fiber alignment and density. Thus, this example of dynamic reciprocity illustrates a positive-feedback relationship: cells collectively generate directional cues (matrix remodeling) that reinforce their own migration (contact guidance, durotaxis), which in turn reinforces the cues [12, 20].

This interplay between mesenchymal-like cell migration and fibrous ECM remodeling is typically observed and studied in the context of large numbers of cells invading matrices over several days and causing large-scale remodeling that is observable even under low magnification (e.g. [10, 13, 22]). However, individual cells are also capable of increasing the alignment and density of ECM fibers locally as they migrate (e.g. [18, 23–27]), although not to the same extent as achieved by masses of cells [28]. Several recent studies support the hypothesis that individual cells are capable of reorganizing the local ECM architecture sufficiently to affect their own migration. Cell-mediated ECM fiber deformations by migratory cells are anisotropic—they are not uniformly distributed around the cell—and are correlated with localized protrusive activity [16, 23, 26, 27, 29, 30]. Anisotropic ECM deformations precede directional protrusions and migration initiation in spreading cells [16], and are correlated with migration direction in migrating cells [26]. Indeed, mesenchymal migration in fibrillar 3D matrices deviates from the persistent random walk (PRW) model (used to describe migration on 2D isotropic substrates) in that migration velocity is biased in the direction of cell alignment [31]. When migratory cells do change directions, this change is preceded by a change in the local ECM strain field [30]. Of course, it is important to recognize that the ECM deformations are being caused by the cell itself as a consequence of its own migration [32]. Therefore, these findings support the existence of a positive-feedback relationship between individual-cell-based matrix remodeling and migration.

In this study, we directly test this self-reinforcing relationship between individual cells and their surrounding ECM by asking how preventing cell-mediated ECM fiber deformation affects cell migration. After validating our photocrosslinked collagen model system, we demonstrate that the ability of individual cells to remodel a fibrous substrate modulates their migration persistence. Substrates with random fibers and reduced remodeling potential cause cells to meander and exhibit decreased persistence in response to a second directionality cue. We further show that gaps in adhesive area between sparse fibers are at least partially responsible for this decrease in persistence. Our findings support the hypothesis that fiber remodeling caused by an individual migrating cell acts as a contact guidance cue that both directs and is reinforced by the cell’s migration.

## Results and discussion

### Crosslinking collagen matrices limits cell-mediated remodeling

To study the role of local ECM remodeling on the migration of individual cells, we utilized HT-1080 human fibrosarcoma cells in photocrosslinkable collagen matrices. HT-1080 cells are commonly used as an *in vitro* model for cancer cell invasion and migrate in an adhesion-based, “mesenchymal” mode through collagen type I matrices except under certain limited conditions [33]. We used a commercially available methacrylated type I collagen that can be crosslinked when exposed to ultraviolet (UV) light in the presence of a chemical photoinitiator (PI) [34]. Because the degree of crosslinking depends in part on UV dose, we titrated UV exposure time using a series of collagen matrices and assessed cell-mediated remodeling by measuring matrix contraction. The combined fiber movements caused by many cells can produce the macroscopic contraction of a floating collagen matrix and the extent of contraction correlates with the cells’ ability to remodel the collagen fibers [22]. To minimize the cells’ exposure to UV light, we sought the lowest exposure time that produced a significant reduction in matrix contraction. UV light was administered using a standard epi-fluorescence microscope with a mercury arc lamp and a UV fluorescence excitation filter (325–375 nm). Paired control matrices, which received the same UV dose, were prepared identically except without PI and were therefore not crosslinked.

**Fig 1:**
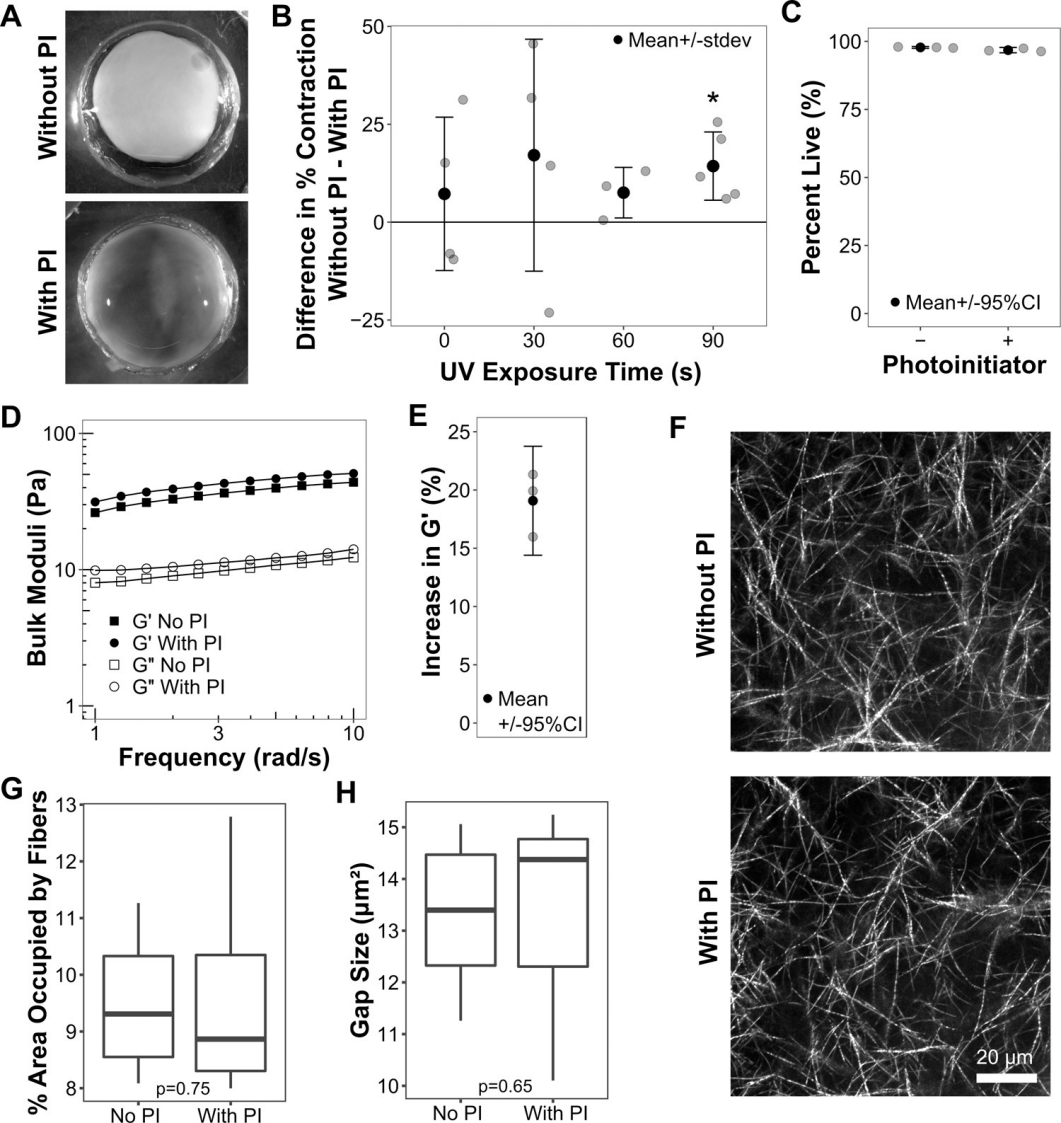
Crosslinking limits the ability of cells to remodel collagen matrices. (A-B) HT-1080 cells were seeded in 2 mg/ml methacrylated type I collagen gels with or without photoinitiator (PI) and then exposed to UV light (325–375 nm, ~7 mW/cm^2^) for 0, 30, 60 or 90 s. Gels were then released from the sides of the well, cultured for 24 h, and finally imaged with a stereoscope. (**A**) Representative images of gels with and without PI (90 s UV exposure time). The gel without PI is visibly contracted relative to the gel with PI as seen by its smaller area and greater opacity. The well diameters in both images are ~ 1 cm. (**B**) Plot of the difference in percent contraction (area) between paired gels with and without photoinitiator (mean +/- stdev). Each point corresponds to a pair of native and crosslinked gels from the same experiment and with identical UV exposure times (left to right, n = 4, 4, 3, and 5 pairs). The only exposure time to yield a significant difference between gels with and without PI was 90 s (p = 0.021, one-sample, two-tailed t-Test). (**C**) Cells were seeded in matrices with and without PI, exposed to UV light for 90 s, incubated 2 h, and then stained with Calcein AM and propidium iodide. Cells exhibiting Calcein staining were classified as “live”; cells exhibiting propidium iodide staining were classified as “dead.” The gray points show the percent of live cells in each experiment; the black points show the mean +/- the 95% confidence interval for each sample type. There was no significant difference between matrices without (“-”) and with (“+”) PI (p = 0.08; t-Test; n = 3 experiments, each with > 200 cells counted).(**D**) Representative rheological data from shear modulus frequency sweep measurements of paired cell-free gels with (circles) and without (squares) PI (90s UV exposure time; 2 h incubation). Storage moduli (G’, black points) and loss moduli (G”, open points) are plotted on log-log axes. The gel with PI had higher storage moduli than the gel without PI at all measured frequencies. (**E**) Gels with PI exhibited higher storage moduli (G’) than their paired native gels in three separate experiments. The percent increase in G’ (with PI—without PI) is plotted for each experiment (gray points) along with the mean (black point) and the 95% confidence interval. The confidence interval does not include 0, demonstrating that the difference in storage moduli is statistically significant. (F-H) Cell-free gels with and without PI (90s UV exposure time; 1.5 h incubation) were imaged in confocal reflectance mode. (**F**) Representative maximum-intensity z-projections from seven independent gels (for each treatment). (**G**) Average fiber-occupied area expressed as a percent of the total field-of-view area. (**H**) Average gap (pore) size. There were no statistically significant differences in fiber density or pore size between gels with and without PI (Kruskal-Wallis test; n = 7, 7).

HT-1080 human fibrosarcoma cells were seeded into paired matrices with and without PI and the difference in percent contraction (change in area) within each pair was quantified (Fig 1A). 90 s was the shortest UV exposure time that produced a significant (p = 0.021) decrease in matrix contraction in matrices with PI relative to those without (Fig 1B). Qualitatively, PI-containing 90 s matrices were also less opaque than matrices without PI, further indicating reduced cell-mediate fiber reorganization (Fig 1A). Lower UV exposure times did not have a significant effect on matrix contraction. Therefore, a 90 s exposure time was used for all subsequent experiments using the methacrylated collagen matrix system.

The viability of cells in matrices with and without PI (90 s UV exposure time) was assessed using the esterase activity (Calcein AM) method (Fig 1C). Cell viability was uniformly high (>96% live cells) and there was no significant difference between matrices without and with PI (p = 0.08).

To directly test the effect of crosslinking on gel mechanical properties, we measured the stiffness of cell-free gels with and without PI using an oscillatory and rotational rheometer. Fig 1D shows storage modulus and loss modulus data from a representative frequency sweep experiment for a pair of gels with and without PI (90 s UV exposure time; 2 h incubation). The average stiffness (storage modulus, G’, at 10 rad/s) of gels without PI was 38.3 +/- 14 Pa, which is within the ranges reported by others [35, 36], and the average stiffness of gels with PI was 45.5 +/- 16 (standard deviation; n = 3 gel pairs). The relatively large standard deviations were likely due to variation in gel handling as the gels were transferred from the dishes, in which they were polymerized and exposed to UV light, to the rheometer. More precise rheological measurements can be obtained by crosslinking the gels directly on the rheometer using an *in situ* UV curing system [34]. Such a system was, however, not available and, if it were, would not necessarily have replicated the conditions used for our cell experiments. Although there was no statistical difference between the average stiffnesses of the gels with and without PI, there was a consistent difference in stiffness within each matched pair. Gels with PI were on average 19% stiffer than their matched non-PI gels (Fig 1E; 95% confidence intervals, 24–14%). The relatively modest increase in stiffness is not surprising given our use of the lowest possible UV dose that produced a measurable physiological response (Fig 1B).

Finally, we examined the fiber architecture of cell-free gels with and without PI to determine whether crosslinking significantly impacted fiber density and pore size. The matrix in both gel types appeared similar (Fig 1F). Quantification of fiber density (Fig 1G) and matrix pore size (Fig 1H) demonstrated no significant differences between gels with and without PI (Kruskal-Wallis test, p = 0.75 and 0.65, respectively). These results agree with those of Gaudet and Shreiber who found no difference in fiber diameter or density using the same system [34]. Taken together, our results demonstrate that our crosslinking procedure increased the stiffness of our collagen gels sufficient to reduce cell-mediated remodeling and did not affect cell viability or fiber architecture. Hereafter, we refer to matrices containing PI as “crosslinked” and those without PI as “native.”

### Crosslinking hinders alignment of fibers by individual cells migrating out of spheroids

According to our hypothesis, in a completely homogeneous and isotropic matrix with no external directionality cues (e.g. chemoattractant gradient), a cell’s migration path should be guided solely by the anisotropies it generates through fiber alignment and condensation. In this hypothetical situation, we predict that a cell’s initial polarization would lead it to deform the matrix fibers along that axis, producing a directionality cue that would propagate in the same direction through the proposed positive-feedback loop. This would result in the cell migrating in a straight line, and one could test the validity of the hypothesis by comparing cells’ paths in matrices with movable and immovable fibers.

However, in the context of an actual matrix produced by the random self-assembly of collagen and seeded with individual cells cultured in chemokinetic media, there are complicating factors. First, although the matrix will be globally isotropic, there will be local anisotropies due to the randomness of fiber self-assembly and the presence of other cells. The propagation of the proposed fiber-based directional cue may be altered when it encounters a conflicting cue, e.g. fibers that are moderately aligned orthogonal to the path or at a locally high density. Second, media with strong, omni-directional migration cues (as opposed to a chemoattractant gradient) promote random migration as cells respond to random fluctuations in local growth factor and chemokine concentrations. Both complicating factors could disrupt a directional migration cue, particularly if that cue were relatively weak. To minimize both complications and to simplify our analysis, we used an alternative approach in which we imposed a non-fiber-based directionality cue on all cells and then assessed the cells’ ability to follow this cue through matrices with movable and immovable fibers.

**Fig 2:**
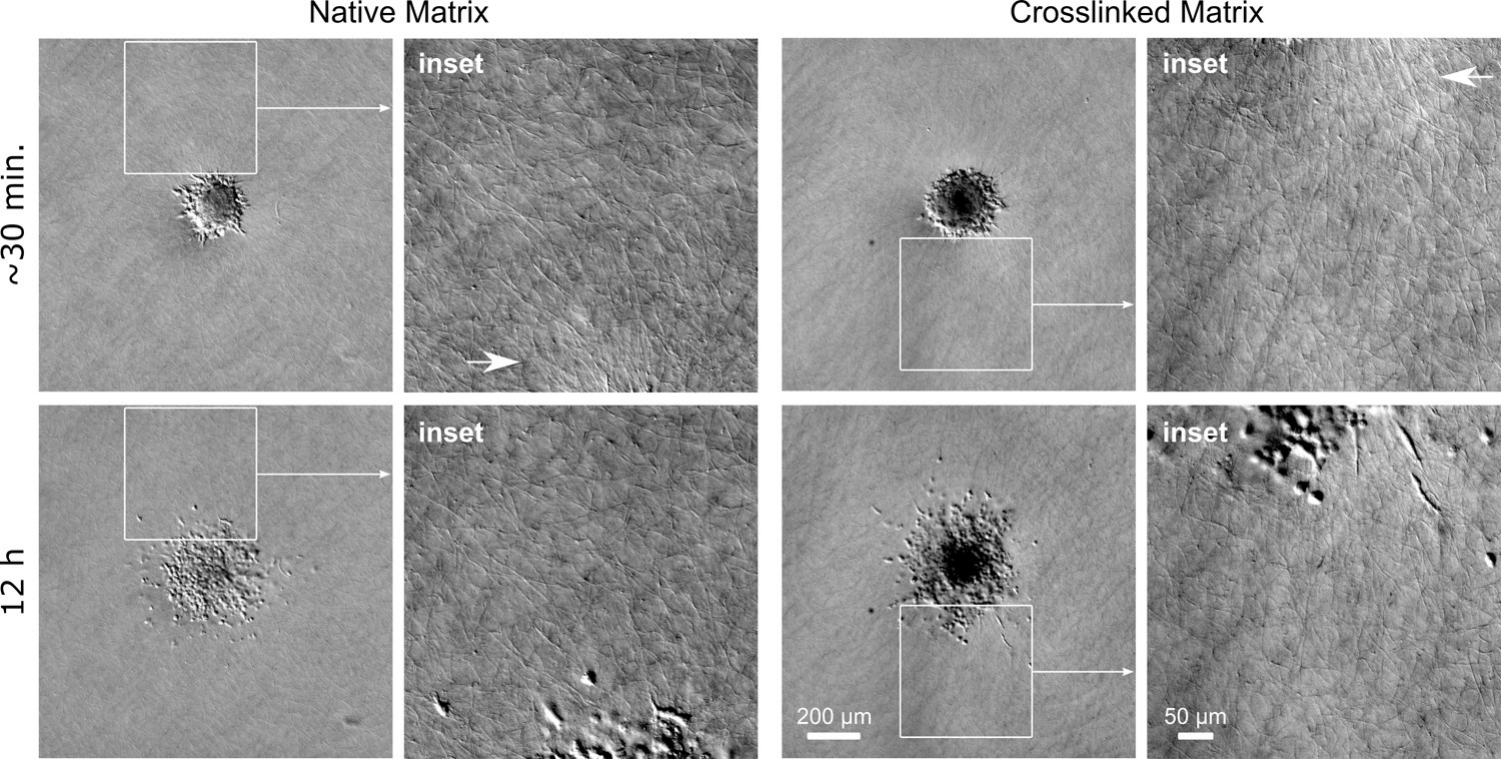
Cells invade native and crosslinked matrices as individuals. Representative DIC images of HT-1080 cells invading from spheroids into native or crosslinked collagen matrices. Images were acquired as soon as possible after seeding (~30 min) and then of the same spheroid at approximately 12 h. Cells invaded radially out of the spheroid as individuals. Fiber alignment was observed in the vicinity of the spheroids in both native and crosslinked matrices (see “inset” images on right; arrows). Scalebar for low-magnification images (left) = 200 μm; scalebar for insets (right) = 50 μm.

We formed HT-1080 cells into spheroids (unorganized, multicellular aggregates) and seeded them in crosslinked and native collagen matrices in a serum-free, migration-promoting medium. Within 30 min after seeding, the cells began to migrate radially outward from the spheroid into the matrix (Fig 2), demonstrating that spheroids induce directed migration, likely through contact inhibition (physical crowding) [12, 14, 37]. The cells migrated individually as has been previously shown for this collagen concentration [37] and not as multicellular strands.

Spheroids and other cell masses are often observed to align the surrounding matrix so that the fibers radiate outward from the mass and act as tracks to guide cell migration (e.g. [12, 13, 28]). Large-scale matrix alignment is typically observed over multiple days and with cells cultured in serum-containing, and therefore contraction-promoting, media. Although we used serum-free media and cultured for relatively short times (< 12 h), we did observe collagen fiber alignment in the vicinity (~100–200 μm) of the spheroids (Fig 2; insets on right; arrows). Interestingly, fiber alignment was observed in both native and crosslinked matrices.

**Fig 3:**
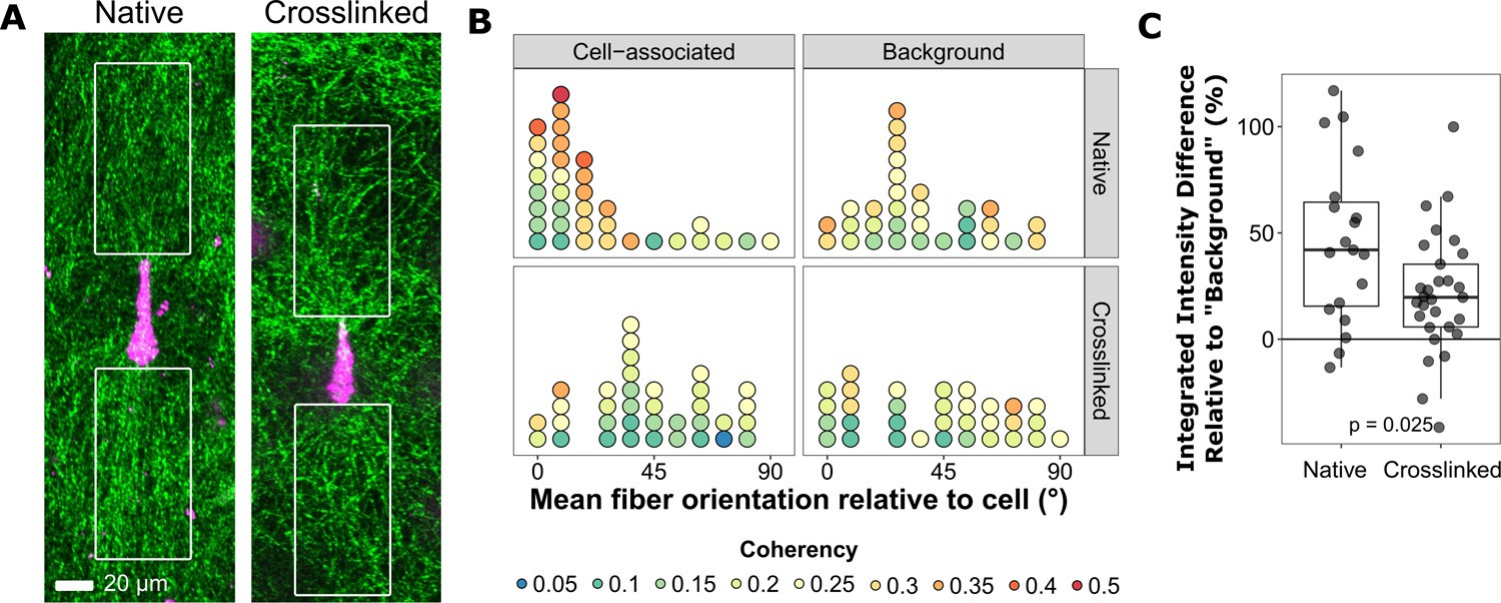
Crosslinking decreases the alignment of fibers associated with individual cells migrating out of spheroids. DiI-stained HT-1080 cells that had invaded from spheroids into native and crosslinked matrices overnight (~12 h) were imaged along with the surrounding fibers to determine the effect of matrix crosslinking on individual cells’ ability to align fibers. We selected cells that were mostly bipolar, mostly parallel to the surface, and not near other cells. (**A**) Representative images of cells (magenta) and collagen matrix (green; reflectance imaging) from native and crosslinked matrices. The images are maximum intensity z-projections of seven slices. Fiber orientation and density were measured within 50x100 μm regions (white boxes) at either end of each cell. Measurements from the two regions were averaged to produce one measurement per cell. (**B**) Plot of predominant orientation in cell-associated regions (“Cell-Associated”) or in random non-cell areas of the same images (“Background”) in crosslinked or native matrices (n = 31 background/native, 32 background/crosslinked, 35 for both cell-associated sets). Each circle represents the measurement from a single cell (average of front/back regions) or non-cell associated area (average of two regions from the same image). Orientations are relative to the long-axis of the cell (parallel to the cell = 0°). Color indicates the coherency—the uniformity of orientations within the region (scale of 0 to 1.0; 1.0 = perfect uniformity). Only the distribution of orientations from the native, cell-associated samples were significantly different from a random distribution (Rao Spacing Test, p < 0.001; all others p > 0.05). The coherency of cell-associated native fibers was significantly different from cell-associated crosslinked (p < 0.001), but not background native (p = 0.25) (Welch’s t-Test; multiple comparisons adjusted using Benjamini-Hochberg). There was no significant difference between crosslinked cell-associated and background fibers (p = 0.25). (**C**) “Density” was measured as the percent difference in the integrated intensity of cell-associated and “background” regions from the same image. Cell-associated regions in native matrices were significantly “denser” than in crosslinked matrices (p = 0.025; two-tailed t-Test; n = 21 native, 28 crosslinked). Data in (B) and (C) come from five independent experiments.

Because our hypothesis centers on the interplay between an individual cell and its local matrix architecture, it was important to determine if cells in our system were responding primarily to their own fiber remodeling and not that of other cells or the spheroid mass. To determine the extent of individual cell-mediated fiber remodeling, we again incubated HT-1080 spheroids in native and crosslinked matrices and imaged individual polarized cells that had invaded the matrix and their surrounding collagen fibers (Fig 3A). We quantified collagen fiber orientation within 50x100 μm regions (Fig 3A, white boxes) on both ends of the cell along its long-axis (“cell-associated”) as well as in two randomly selected, cell-free areas within the same image (“background”) (Fig 3B). The orientation analysis measured the predominant direction of alignment (“orientation”) as well as the uniformity of alignments within each region (“coherency”). For example, if a region has an orientation of 0° and a coherency of 1.0, the fibers within this region are all uniformly parallel to the long-axis of the cell. Each circle in Fig 3B represents data from one cell; its position on the x-axis indicates the orientation, while its color indicates its coherency. Fibers associated with cells in native matrices were predominantly oriented parallel to the cell (Fig 3B; Rao Spacing Test, p < 0.001). In contrast, the distribution of fiber orientations associated with cells in crosslinked matrices or in background areas of either matrix type were not significantly different from a random distribution (Rao Spacing Test, p > 0.05). Moreover, the coherency of cell-associated fibers in native matrices (0.235 +/- 0.1) was significantly higher than that of crosslinked matrices (0.163 +/- 0.07) (p < 0.001, t-Test). There was no significant difference in coherency between cell-associated and background fibers (0.213 +/- 0.08) in native matrices; however it is important to note that the high coherency values with cell-associated fibers were predominantly associated with low fiber angles whereas the distribution was more uniform for background fibers. These data suggest that, at least on a local scale, matrix fiber orientations are random except in close vicinity to cells in native matrices.

To corroborate this observation, we measured integrated intensities within these same 50x100 μm cell-associated regions as a rough determination of matrix density differences in native and crosslinked matrices. Because intensity values can be highly variable and therefore difficult to compare between images, integrated intensities from cell-associated regions were expressed as percent differences relative to the average integrated intensity from non-cell-associated regions in the same image (Fig 3C). The matrix associated with cells in native matrices was on average 45% more dense than non-cell-associated areas. This is significantly different from crosslinked matrices (p = 0.025), which showed only a 20% increase in matrix density. Taken together, these data indicate that cells in native matrices increase the local alignment and density of matrix fibers significantly relative to cells in crosslinked matrices. These findings support our use of this model system to study how a cell’s self-generated local matrix remodeling affects its migratory behavior.

### Local cell-mediated fiber alignment promotes migration persistence

**Fig 4:**
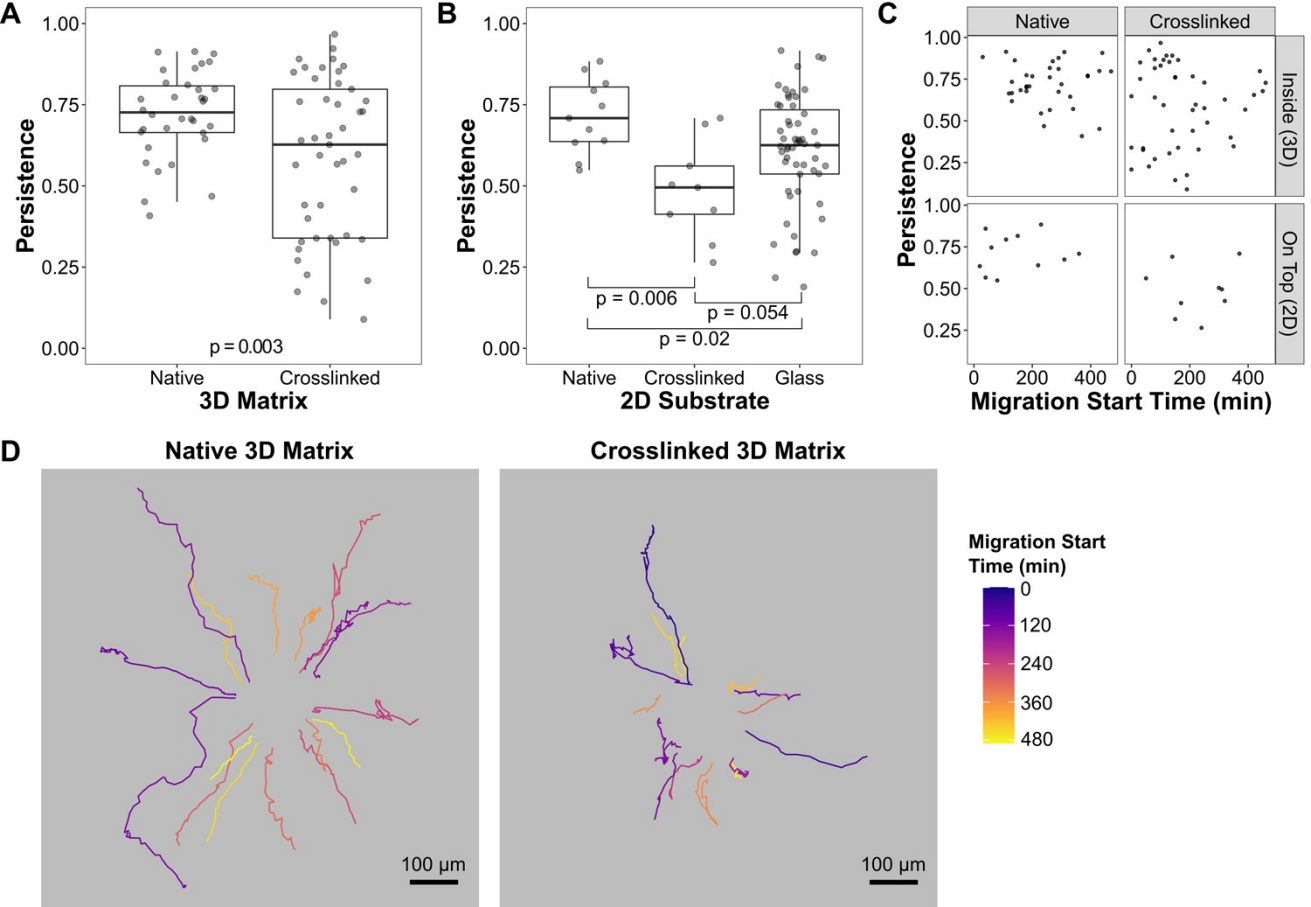
Matrix crosslinking decreases cell migration persistence. Cell emigration out of spheroids was tracked over time. For persistence calculations, tracks were truncated to four hours. (**A**) The average persistence of cells in native matrices is significantly higher than that of cells in crosslinked matrices (p = 0.003; two-tailed t-Test; n = 34 native, 45 crosslinked cells). Box-and-whisker plot: dark line indicates median; box encompasses 25th-75th percentiles; whiskers extend to the largest value no larger than 1.5 times the box width (interquartile range). Each dot represents data from an individual cell from one of four independent experiments. (**B**) Identical experiment to (A) except that spheroids were seeded on top of native and crosslinked matrices rather than embedded within them. Cells on native matrices were more persistent than those on crosslinked matrices (p = 0.006; two-tailed t-Test; n = 11 native, 9 crosslinked). The average persistence of cells migrating out of spheroids onto glass coverslips was in between that of cells on the matrices, significantly different from native (p = 0.02), but only marginally significant from crosslinked (p = 0.054). P-values were adjusted for multiple comparisons using the Benjamini-Hochberg (false discovery rate) method. Data are from three independent experiments. (**C**) Persistence data from (A; “inside”) and (B; “on top”) plotted as a function of the time at which the cell began to be tracked. Cells exited the spheroid throughout the 12 h incubation and tracking began as soon as they could be distinguished from the spheroid. Time 0 is the beginning of the timelapse recording, not the time of seeding. There is no correlation between persistence and time of track initiation for any of the four datasets (Spearman’s Rank Correlation: crosslinked-inside, rho = -0.06, p = 0.72; native-inside, rho = 0.01, p = 0.95; crosslinked-ontop, rho = 0.08, p = 0.84; native-ontop, rho = 0.26, p = 0.45). (**D**) Migration paths of cells from representative native and crosslinked movies (same source data as panel A). The paths are color-coded based on the time at which the cell began to be tracked. Paths begin at the center. Cell paths are mostly distinct, although short sections of path overlap are occasionally observed. Scale bar, 100 μm.

Based on our positive-feedback hypothesis, we predicted that individual cells migrating outward from a spheroid would continue to migrate in their original direction, provided that they were able to align matrix fibers as they migrated. To test this prediction, we performed time-lapse tracking of cells emigrating from spheroids into native and crosslinked matrices. Similar to our earlier experiments, crosslinked and native matrices only differed in whether they contained PI. We calculated persistence from four-hour tracks of individual cells as a measure of directional cell migration. The median persistence was significantly reduced (p = 0.003) in cells seeded in crosslinked matrices compared to cells in native matrices (Fig 4A). Although HT-1080 cells are capable of amoeboid migration and proteolytic collagen fiber degradation, previous work has shown that collagen matrices of this density (2 mg/ml) do not require proteolytic degradation for cells to migrate and do not trigger amoeboid migration, even in the presence of matrix-metalloproteinase inhibitors [37]. Accordingly, we did not observe amoeboid migration or obvious proteolytic degradation (e.g. tunnel formation).

The reduced persistence of cells in crosslinked matrices may have been due to a reduced ability to align fibers, as we hypothesized, but may have also resulted from increased steric hindrance. A matrix with fibers that resist stretching may prevent a cell from passing through a narrow gap between fibers, and instead cause a change in path and a reduction in persistence. To investigate this second potential cause, we repeated the previous experiments, but seeded spheroids on top of native and crosslinked matrices. Only cells migrating on the surface of the matrices were imaged. Cells migrating on top of a matrix would still be affected by a reduced ability to align fibers, but would not be constrained by the need to pass through matrix pores. Similar to the embedded spheroid experiments, we found that the median persistence was significantly reduced in crosslinked matrices relative to the native gels (Fig 4B; p = 0.006). Therefore, crosslinking-induced steric hindrance was not responsible for our observations of cells embedded in 3D matrices (Fig 4A). A related concern is that a previous report found that collagen matrices crosslinked using the same method as ours exhibited lower susceptibility to collagenase digestion [34], calling into question whether crosslinked and native fibers would be equally cleaved by matrix-metalloproteinases. Although we did not observe signs of proteolytic degradation (e.g. tunnel formation) or amoeboid migration in the 3D experiments, the 2D control experiments also obviate the impact of any potential difference in cell-mediated proteolysis. Furthermore, this experiment demonstrates that the effect of fiber remodeling on cell migration is not limited to 3D matrices, but is instead a general property of fibrous substrates.

Notably, cells migrating out of a spheroid onto fibronectin-adsorbed glass (Fig 4B) had a lower median persistence than cells on native collagen matrices (native = 0.709 vs. glass = 0.625; p = 0.02). This agrees with Wu et al. [31] who observed that HT-1080 cells in collagen matrices exhibited straighter migration paths than when cultured on glass. It supports the hypothesis that cell-mediated fiber alignment and condensation provide an additional directionality cue above that of the spheroid. While the strength of the contact inhibition imposed by the spheroid [12, 14, 37] should diminish as the cells disperse, a self-generated matrix remodeling cue would not. Taken together, these findings support the hypothesis that self-reinforcing matrix fiber remodeling by individual cells promotes persistent migration.

Because cells in native matrices can align and condense fibers, we asked whether the increased persistence of cells in these matrices was due to earlier cells establishing tracks of aligned fibers for later cells to follow. If this were so, we would expect that cells emigrating out of the spheroid at later timepoints would have a higher persistence than the initial “pioneer” cells. However, there is no correlation between persistence and time of migration initiation for cells migrating on native or crosslinked matrices (Fig 4C). Moreover, while cell migration paths occasionally show partial overlap, cells in both native and crosslinked matrices predominantly follow their own unique path (Fig 4D). These findings agree with Haeger et al. who showed that HT-1080 cells in similar density collagen matrices do not exhibit a leader-follower pattern of migration and instead use individualized paths [37]. The observation that later-migrating cells did not have higher persistence also argues against the influence of progressive matrix alignment caused by the spheroid as a whole. Instead, taken together, our results suggest that the ability of individual cells to align fibers locally increases their migration persistence. This agrees with recent findings by Doyle and co-workers [26] who demonstrated a correlation between anterior matrix deformation and cell migration persistence.

### Inflexible randomly oriented fibers decrease persistence through adhesive area discontinuity

The previous experiments demonstrate that cells in native collagen matrices remodel their local fiber structure resulting in increased migration persistence. Increased fiber alignment and density increases local stiffness, thereby promoting directional migration via contact guidance and durotaxis [6, 18–20]. Crosslinking the collagen matrices reduces the ability of cells to create both gradient types. Crosslinking also increases the global stiffness of the matrix, yet median persistence on crosslinked matrices was lower than both the (more compliant) native matrix and the (stiffer) glass substrate (Fig 4B). Cells on the crosslinked matrices not only did not benefit from the remodeling/migration feedback system of cells on the native matrices, but were also hindered from taking full advantage of the contact guidance cue imposed by the spheroid as exhibited by the cells on glass.

Earlier work by us and others [14, 15, 18, 24, 38] has demonstrated that substrate features that allow linear, stepwise formation of adhesions promote persistent cell protrusions, leading to local cell polarization. If the adhesive features of the substrate are aligned in a single direction (e.g. aligned fibers), cell protrusions are also directed in the same direction, resulting in cell polarization. In contrast, if the features are oriented randomly, cell protrusions will not be directed uniformly and will prevent or limit the cell from maintaining a consistent polarization in any one direction. This phenomenon necessarily impacts migration direction and therefore persistence.

We therefore hypothesized that random, crosslinked fibers were causing cells to deviate from the most direct path away from the spheroid by diverting cell protrusions based on local fiber orientations. We predicted that migration persistence should be highest when there are no substrate directionality cues that compete with cell emigration from the spheroid. A completely isotropic substrate such as fibronectin-adsorbed glass would not provide any conflicting cues. We further predicted that as random fiber density approaches that of an isotropic substrate, cell persistence should increase because the gaps between fibers become small enough that sequential adhesion formation can span them and/or because the migration path deviations caused by the random fibers are smaller. These predictions are supported by studies showing a correlation between 3D matrix fiber density and cell spreading and persistence [12, 39].

**Fig 5:**
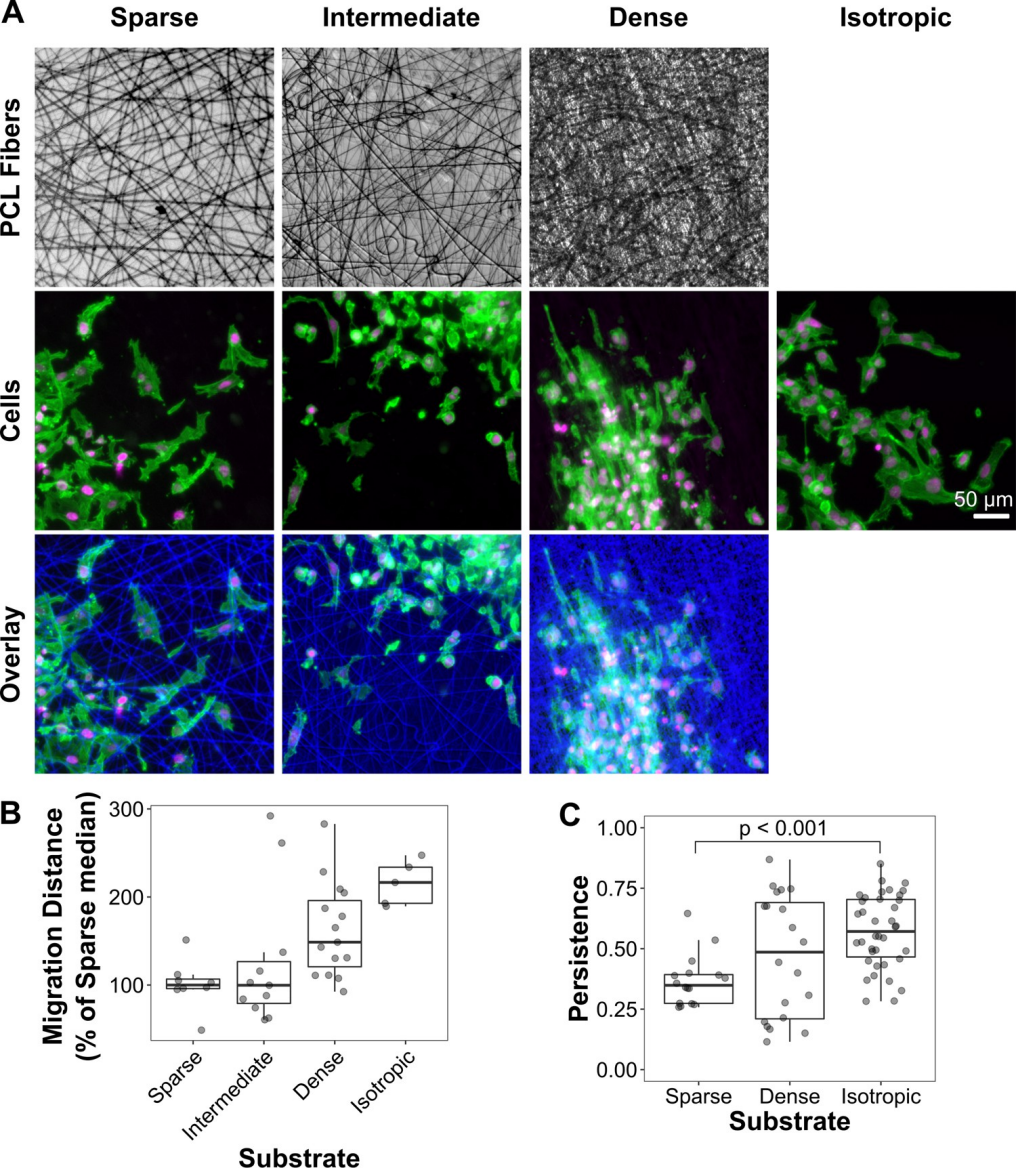
Fiber density affects migration persistence. Spheroids were seeded on top of PCL substrates of all three fiber densities as well as on plastic well-plates (B) or glass coverslips (A, C) (both labeled “isotropic”). (**A**) Representative images of cells migrating out of spheroids onto PCL scaffolds or glass (“isotropic”) imaged 12 h post-seeding. Brightfield images of the PCL fibers are shown in blue in the overlay. Nuclei (DAPI) are labeled magenta; cell bodies (phalloidin-stained actin) are labeled green. Scalebar = 50 μm. (**B**) After culturing for 24 h, the cells were imaged and their distance from the original spheroid boundary was calculated. Each point represents the median cell migration distance for a single spheroid, expressed as a percentage of the overall median of all spheroids from the Sparse samples. Box-and-whisker plot: dark line indicates median; box encompasses 25th-75th percentiles; whiskers extend to the largest value no larger than 1.5 times the box width (interquartile range). Each dot represents data from an individual cell. All groups are significantly different from the others (p < 0.05) except for Intermediate-Isotropic (p = 0.06) and Intermediate-Sparse. Wilcox Rank-Sum Test; p-values adjusted using the Benjamini-Hochberg (false discovery rate) method; from left to right, n = 8, 11, 15, 5. Data were collected from three independent experiments. (**C**) In separate experiments, spheroids were seeded on Sparse, Dense, and glass (Isotropic) substrates and imaged over time as in Fig 4. (Note that the Isotropic data are the same data as the glass sample in Fig 4B.) Each point represents the persistence of a single cell. The persistence of cells on Sparse and Isotropic substrates are significantly different (p < 0.001). The average persistence of cells on Dense substrates is intermediate between the other two, but not significantly different from either (p = 0.12). Welch’s two-tailed t-Test; p-values adjusted using the Benjamini-Hochberg (false discovery rate) method; from left to right, n = 16, 20, 38. Data were from four independent experiments.

To test our predictions, we seeded spheroids onto 2D electrospun polycaprolactone (PCL) scaffolds with randomly oriented fibers in a range of densities and isotropic (glass or plastic) substrates (Fig 5A). PCL scaffolds have a stiffness of several MPa [40], which is orders of magnitude greater than that of type I collagen matrices (Fig 1D and Refs. [34–36]). Individual PCL fibers are too stiff for cells to move and the scaffolds are thin and bonded to a plastic coverslip, therefore, the cells cannot align or otherwise remodel the fibers and do not sense any differences in stiffness between the various substrates [41]. By using a reductionist stiff-fiber, 2D model system, we avoided two problematic side-effects of altering 3D matrix density: altering matrix stiffness and porosity.

The median cell migration distance for each spheroid was measured after 24 hours (Fig 5B). Increasing the density of PCL fibers increased the median migration distance (sparse vs. dense, p<0.001); providing cells with an isotropic surface increased the distance even further (dense vs. isotropic, p<0.05). These data suggest that sparse, randomly oriented fibers impede directed migration while high fiber densities and isotropic (featureless) substrates support it.

To further test our predictions, we repeated the experiments but collected timelapse movies of cell migration and calculated persistence values for individual cells (Fig 5C; 6.5 h time period). The intermediate density scaffold was not included because the median migration distances were similar to sparse (Fig 5B). We measured a trend of increasing persistence values with increasing adhesive area density. Although only the sparse and isotropic populations were statistically different (p<0.001), the dense population was clearly intermediate between these two extremes. Our results agree with those presented by Jana et al. who demonstrated that cells on a bi-directional grid of polymer fibers increase their elongation, speed, and persistence with increasing fiber density [42].

Because the PCL fibers have larger diameters and greater inter-fiber spacing than the collagen matrices used in the preceding experiments, we expect some differences between the behavior of cells on the two substrate types. For example, the larger inter-fiber spacing should exaggerate cell meandering, resulting in decreased persistence [42] relative to the more closely-spaced fibers in collagen matrices. Although challenging to prepare, 2D crosslinked collagen matrices with a range of fiber densities, but constant stiffnesses and fiber diameters would provide a more direct comparison to our 3D matrix studies. Based on the PCL experiments (Fig 5), we would expect an increase in collagen fiber density in such matrices to increase cell persistence, approaching that of cells on glass/isotropic substrates (Fig 4B), as cells encountered fewer adhesive area gaps. In contrast, a decrease in collagen fiber density would decrease persistence as cells were forced to follow fibers around gaps in adhesive area.

Taken together, our observations support the hypothesis that cells in crosslinked matrices of randomly oriented fibers migrate less persistently, and this is due in part to a discontinuous adhesive area caused by conflicting fiber orientation cues. Cells in matrices with flexible fibers can increase local fiber alignment and condensation, thereby increasing their persistence by increasing local adhesive area continuity. Such cell-mediated remodeling would also lead to a local increase in matrix stiffness, which could also promote persistence [19, 26]. However, the above results from PCL substrates in which fiber density was altered independent of stiffness demonstrate that the fiber structure of the substrate alone is able to modulate cell migration.

## Conclusion

This study demonstrates the dependence of an individual cell’s migratory behavior on its ability to manipulate its surrounding environment. Cells in environments with deformable fibers can produce their own contact guidance cues through fiber alignment and condensation, leading to a positive-feedback relationship between matrix remodeling and migration. In contrast, cells in environments with random, immobile fibers face conflicting fiber orientations that produce discontinuous adhesion areas for cell protrusions [18], leading to less persistent migratory paths. Indeed, inflexible, random fibers attenuated the cells’ ability to follow a second directionality cue (emigration out of a spheroid).

While the relationship between large-scale cancer cell invasion and matrix remodeling has been well-described [10, 13, 22], our work shows that individually migrating cells use the same strategy to guide their own migration. Our results support other recent studies that have demonstrated the effects of matrix deformation on protrusion direction and cell migration [16, 26, 30]. In addition, our work adds to the growing body of research demonstrating how the canonical mesenchymal migration process described for cells on hard, 2D, featureless (isotropic) substrates must be adapted for more complex and physiologically relevant environments. Finally, this knowledge provides us with a more nuanced understanding of how to manipulate cell migration—both to inhibit (e.g. in the context of cancer cell invasion) and to stimulate (e.g. in engineering tissue scaffolds that promote host cell invasion) [3].

## Materials and methods

### Cell culture

HT-1080 human fibrosarcoma cells (purchased directly from ATCC; item CCL-121) were cultured in MEM + Earl’s Salts, L-glutamine, 10% fetal bovine serum (FBS), and non-essential amino acids in a 5% CO2 incubator. Unless otherwise specified, cells in experiments were cultured in CCM1 (Hyclone, Thermo Fisher Scientific), a CO_2_-independent and serum-independent medium. All cell-culture reagents were from Thermo Fisher Scientific unless otherwise indicated.

### 3D methacrylated collagen matrix production and crosslinking

Methacrylated bovine type I collagen matrices were produced using a kit from Advanced Biomatrix (PhotoCol; catalog #5201; lots 7649 and 8479). The percent methacrylation is reported by the manufacturer to be >20%. The mixture for each matrix was produced individually at a final volume of 300 μl and a final concentration of 2 mg/ml. Crosslinked and native matrices were identical except that the crosslinked matrices contained 1 mg/ml Irgacure photoinitiator (PI). Each matrix solution was pipetted into a homemade 1 cm well dish (described below) and then incubated at 37°C for 15 min to facilitate gel polymerization. All matrices were then irradiated with 325–375 nm light (transmission of the emission filter; 7 mW/cm^2^ measured at the sample) using a Nikon TE2000 widefield inverted microscope with a PlanApo 4x/0.2NA objective for a range of times (Fig 1) or 90 s (Figs 2–4). The matrix was in focus and centered in the field-of-view at the time of irradiation. Additional medium was added on top of the collagen matrices and the samples were returned to the incubator or transferred to a stage-top incubator for timelapse imaging. Medium type and incubation duration depended on the exact experiment.

Matrices were cast in homemade polydimethylsiloxane (PDMS) rings (1 cm inner diameter) that were attached to the center of 35 mm glass-bottomed dishes (Matsunami or Mat-Tek). The purpose of the rings was to reduce the volume of the dishes. Rings were produced by mixing a 1:10 mass ratio of curing agent to base (Sylgard 184, Dow), degassing, pouring the solution into a petri dish to a depth of ~1 cm, curing overnight at 60°C, and then cutting-out rings with a 1 cm inner diameter. The rings were sterilized by immersing in 70% ethanol and then drying in a laminar flow hood.

### Macroscopic gel contraction and cell viability experiments (Fig 1)

Methacrylated collagen matrix solutions were prepared as stated above and included 1.0 x 10^5^ HT-1080 cells per matrix. Samples were produced in matched pairs with and without PI for each UV exposure time (0, 30, 60, and 90 s). After irradiation, complete media (serum-containing) was added to the samples and then the matrices were released from the sides of their wells by running a thin metal spatula around the edges. Samples were incubated for 24 h and then imaged using a Zeiss SV6 Stemi Stereoscope with an Insight 2 camera, run by SPOT imaging software. The initial matrix area was assumed to be equal to the inner area of the PDMS ring. Both the initial and final areas of the matrix were measured by hand tracing using FIJI/ImageJ. The percent change in area was calculated for each matrix [(A_initial_—A_final_)/A_initial_ x 100%]. Because there was variation in percent changes values between experiments, we calculated the difference in percent change between matched pairs of matrices with and without PI for each UV exposure time and each experiment. These are the values that are plotted in Fig 1B. The null hypothesis is that there is no difference in percent contraction between the matrices with and without photoinitiator; i.e. that the population mean (μ) is equal to 0. The null hypothesis was tested using a one-sample, two-tailed t-Test with Welch’s modification for unequal variances.

Cell viability (Fig 1C) was assayed in cells seeded in matrices with and without PI in CCM1 media (90 s UV exposure time) and incubated for 1.5 hours. Samples were stained with 1 μM Calcein AM (Corning, 354217) and 0.5 μg/ml propidium iodide (Calbiochem, 537059) in CCM1 for 30 min and then immediately imaged. Five randomly selected fields-of-view were imaged per sample with a Nikon Eclipse Ti-2 microscope, Plan Fluor 10x/0.30NA objective, and a Hamamatsu ORCA Flash-4.0 V3 sCMOS monochrome camera (0.65 μm/px). Propidium iodide was excited with a 550/15 nm LED (Lumencor, Spectra-X) and imaged using a 641/74 nm emission filter. Calcein was excited with a 470/24 nm LED and imaged using a 525/50 nm emission filter. Cells with Calcein fluorescence were counted as live; cells with propidium iodide fluorescence were counted as dead. Measurements were performed automatically using a custom-written FIJI/ImageJ macro involving segmentation with a computer-defined threshold and watershed function. Statistical differences were assessed using a two-tailed t-Test.

### Rheological measurements (Fig 1)

Methacrylated collagen gels with and without PI were prepared as described above, except without cells (CCM1 medium only) and with a total volume of 1.5 ml. 90 s UV irradiation was conducted as described above and the gels were incubated for two hours before being gently transferred to the rheometer. The shear storage and loss moduli were measured using an oscillatory and rotational rheometer (Anton-Paar MCR302) with a 25 mm sandblasted parallel-plate measuring geometry and a sandblasted bottom plate. Sample temperature was kept at 37°C and a measuring gap of 0.6 mm was used. Frequency sweeps were conducted from 1–100 rad/s with a strain of 0.5%. Gels with and without PI were prepared simultaneously and measured consecutively. Measurements for both gel types often became unreliable at higher angular frequencies as evidenced by erratic variations in storage and loss moduli. Frequency sweeps were therefore truncated to 1–10 rad/s. The percent difference in storage moduli of paired native and crosslinked samples was calculated for each of the three independent trials and plotted in Fig 1E. The 95% confidence interval for these measurements does not include 0, demonstrating that the percent difference is significantly different than 0.

### Matrix structure characterization (Fig 1)

Methacrylated collagen gels with and without PI were prepared as described above, except without cells (CCM1 medium only) and with a total volume of 100 μl. 90 s UV irradiation was conducted as described above and the gels were incubated for 1.5 hours before imaging. Fiber images were acquired on a Nikon TE2000 C2si laser scanning confocal microscope with a PlanApo VC 60x/1.4NA objective, PMT detectors, single-direction scanning, and a 20 μm pinhole (optimized for the collagen channel) in reflectance mode (ex. 515 nm Argon Ion laser; em. 525/50 nm). 2-μm-thick z-stacks were acquired with a pixel size of 100 nm/pixel and a z-step of 300 nm. Seven matched pairs of native and crosslinked matrices were imaged and analyzed. Three to five images were acquired per sample. Prior to analysis, maximum-intensity projections of each z-stack were processed with a median filter (1-pixel radius). Fiber density and gap size analysis were performed with the TWOMBLI (version 1) ImageJ analysis package [43]. Parameters used: Contrast Saturation, 0.35; Min Line Width, 10; Max Line Width, 20; Min Curvature Window, 40; Max Curvature Window, 40; Minimum Branch Length, 10; Maximum Display HDM, 250; Minimum Gap Diameter, 0.34. Fiber area measurements were averaged within each sample and reported as a percent of the total field-of-view area (~11,000 μm^2^). Gap size measurements were also averaged within each sample. Differences were assessed with the Kruskal-Wallis test.

### Spheroid formation

Low melting temperature agarose powder (Sigma, A9414) was dissolved in water at 4 mg/ml, sterilized by autoclaving, and stored at 5°C in aliquots. The stock agarose was warmed to 70°C in a heatblock and diluted with warm sterile PBS to 2 mg/ml. 50 μl of the diluted agarose was pipetted into the wells of a flat-bottomed 96-well plate (Greiner) and allowed to solidify at room temperature for 15 min. 1 x 10^4^ HT-1080 cells in complete growth media were added to each well and allowed to incubate at 37°C for 48 h. For some experiments, the cells were labeled with DiI (ThermoFisher) prior to seeding. Spheroids were screened at low-magnification; those that were irregularly shaped or abnormally small or large were not used.

### Collagen fiber alignment and density imaging and analysis (Figs 2 and 3)

Spheroids were embedded in collagen matrices by mixing them with the unpolymerized collagen solution. Only a small number (3–5) of spheroids were seeded per sample. After UV irradiation, CCM1 was added to each matrix and all samples were either transferred to the cell culture incubator or to a microscope stage-incubator for timelapse imaging.

DIC images in Fig 2 were acquired with a Nikon Eclipse Ti-2 microscope, Plan Fluor 10x/0.30NA objective, and a Hamamatsu ORCA Flash-4.0 V3 sCMOS monochrome camera (0.65 μm/px). The displayed images were flat-field corrected. Fiber alignment z-stack images in Fig 3 were acquired on a Nikon TE2000 C2si laser scanning confocal microscope with a PlanApo 10x/0.45NA objective, PMT detectors, single-direction scanning, and a 20 μm pinhole (optimized for the collagen channel). Collagen fibers were imaged in reflectance mode (ex. 515 nm Argon Ion laser; em. 525/50 nm). Cells that were stained with DiI prior to spheroid formation were imaged using: ex. 561 nm solid-state laser; em. 600/50 nm. Pixel sizes ranged from approximately 0.3 to 0.6 μm/px and the z-step size ranged from 1–3 μm.

The orientation of cell-associated matrix was measured in samples that had been incubated overnight (~12 h). Throughout the analysis process, we used the cell (DiI) channel and not the matrix channel to select cells and draw regions. We imaged and analyzed cells that were mostly bipolar, mostly parallel to the surface, and not near other cells. Using FIJI/ImageJ, we identified the z-slice in which the cell was most in-focus and then generated a maximum intensity z-projection of the slices -3 to +3 that slice. We then rotated the projection image (with bilinear interpolation) so that the cell was horizontal. A 50x100 μm region (e.g. white boxes in Fig 3B) was drawn at one end of the cell and OrientationJ [44] (with Laplacian of Gaussian sigma = 2.0) was used to measure the orientation and coherency of the matrix channel within the region. The process was repeated for the other side of the cell. In a few cases, a region had to be drawn slightly smaller because the cell was too close to the edge of the image. Non-cell-associated (“background”) areas were selected from the same rotated projection images, but by a different analyst who did not know which cells had been measured. The analyst selected two 50x100 μm regions that were as far away as possible from cells. As in the previous analysis, all region selection decisions were made based on the cell, and not the matrix, channel. The background regions were analyzed in the same manner using OrientationJ. The two orientation values for each cell (i.e. from either side) were averaged (circular mean) as were the coherency values (arithmetic mean). The same process was applied to the pairs of background measurements. The distribution of orientations for each sample type were compared to a random/uniform distribution using the Rao Spacing Test using the R package, ‘circular’ (https://rdocumentation.org/packages/circular/versions/0.4-93). Differences in coherency were tested using a two-tailed t-Test with Welch’s modification for unequal variances and p-values were adjusted for multiple comparisons using the Benjamini-Hochberg (false discovery rate) method.

Matrix “density” was measured as the integrated intensity of the cell-associated 50x100 μm regions described above, normalized by the integrated intensity averaged from three cell-free regions. The analysis used the same rotated projection images that were used in the orientation analysis, however some images were excluded because they contained saturated areas. Cell-associated and background regions were selected as in the orientation analysis and based on the cell channel only (matrix channel was hidden). In some cases, a region had to be drawn slightly smaller because the cell was too close to the edge of the image; therefore, all integrated intensity measurements were normalized to region area. The two integrated intensity values for each cell (i.e. from either side) were averaged, as were the three background values from each image. Each cell-associated mean integrated intensity measurement was expressed as a percent difference relative to the mean associated background measurement [(I_cell_—I_background_)/I_background_ x 100%]. The differences between the two populations were assessed using a two-tailed t-Test with Welch’s modification for unequal variances. In this analysis, a single outlier point, which had a value > 99.999th percentile, was excluded.

### Timelapse migration in 3D collagen imaging and analysis (Fig 4)

Crosslinked and native matrices containing spheroids with DiI-labeled cells were prepared as described above. After UV irradiation, CCM1 media was added to each sample and the samples were transferred to the microscope for timelapse imaging (see below). For experiments in which spheroids were seeded on top of matrices (Fig 4B), cell-free collagen matrices were produced and incubated for 20 min as in the procedure above. Spheroids in CCM1 were then pipetted onto the top of the polymerized matrices. The samples were then UV irradiated and transferred to the microscope for timelapse imaging. The glass substrate data for Fig 4B are the same data as the isotropic data in Fig 5C; see the following section for experimental details.

Timelapse imaging was performed on two different microscopes that acquired essentially the same data. System #1: A Nikon Eclipse Ti-2 inverted microscope with a PlanFluor 10x/0.3NA objective (sometimes with a 1.5x magnification insert) and a Hamamatsu ORCA-Flash 4.0 V3 sCMOS monochrome camera (pixel resolution = 0.65 μm/px or 0.43 with 1.5x insert). Samples were maintained at 37°C (CCM1 media is CO_2_-independent) with a Tokai-Hit stage-top incubation chamber. DiI fluorescence was excited with a 550/15 nm LED (Lumencor, Spectra-X) and emission was collected with a 641/74 nm emission filter. System #2: A Nikon TE2000 inverted microscope with a PlanApo 4x/0.2NA or a 10x/0.45NA objective and a Hamamatsu ORCA-Flash 4.0 V3 sCMOS monochrome camera (1.61 μm/px for 4x, 0.645 μm/px for 10x). Samples were maintained at 37°C with a stage-mounted dish incubator (Warner Instruments, DH-35). DiI fluorescence was excited with a mercury arc lamp using a 545/22 nm excitation filter and the emission was collected with a 605/70 nm emission filter. Regardless of the microscope, the focus was positioned near the equator of the spheroid. Images were acquired every 10 min for up to 12 h. Both microscopes contained a Perfect Focus System (PFS) to maintain focus during the timelapse imaging.

The resulting movies were imported into FIJI/ImageJ for analysis. If x-y drift was observed, it was corrected using the Linear Stack Alignment with SIFT plugin (https://imagej.net/plugins/linear-stack-alignment-with-sift). Individual cells were tracked using the ImageJ Manual Tracking plugin. A cell’s track was initiated at the earliest timepoint it was distinguishable from the spheroid and other cells. With the exception of cells that migrated out of focus prematurely, cell tracks were terminated at the end of the timelapse movie. Persistence was calculated as the cell’s total migration path length divided by the straight-line distance between its starting and ending points (net displacement). Because the track duration can affect this calculation, all tracks were truncated to 4 h and those tracks with shorter durations were excluded from the analysis. Statistical comparisons in Fig 4A and 4B were performed using two-tailed t-Tests with Welch’s correction for unequal variances. P-values were adjusted for multiple comparisons using the Benjamini-Hochberg (false discovery rate) method. Correlations in Fig 4C were assessed using the non-parametric Spearman Rank Correlation test.

### Migration on PCL and Isotropic substrates imaging and analysis (Fig 5)

2D scaffolds with random 700 nm diameter electrospun polycaprolactone (PCL) fibers, mounted on 15 mm diameter plastic coverslips were purchased from Nanofiber Solutions (NanoECM 24-well plate inserts, item 24201). The “sparse” fiber density is their standard density (20 μm thick); the “intermediate” and “dense” fiber densities were custom ordered (same item number, 24201) as “40 micron density” and “100 micron density,” respectively. The PCL substrates were held in 12-well cell culture plates (Grenier) to facilitate culturing, staining, and imaging. Empty 12-well plate wells served as the “Isotropic” substrates for the experiments in Fig 5B. Isotropic substrates for Fig 5C were glass-bottomed dishes (MatTek or Matsunami; No. 1.5 coverslips). All substrates were adsorbed with 5 μg/ml human plasma fibronectin (Corning) in PBS for 30 min at room temperature or overnight at 5°C. The substrates were rinsed once with PBS before seeding spheroids. Spheroids were seeded onto the substrates, allowed to attach (~30 min), and then the media was replaced with CCM1.

For the migration distance experiments (Fig 5A and 5B), samples were imaged (brightfield only) after the initial media exchange to measure the starting size of the spheroid. The initial imaging was performed directly in the 12-well plates in which the samples were kept. After imaging, the samples were returned to the cell culture incubator for 24 h. Samples were then fixed with 4% formaldehyde, permeabilized with 0.5% Triton X-100, and blocked with 4% BSA. The samples were stained with 5 μg/ml DAPI (Sigma) in PBS with 2% BSA. Scaffolds were then imaged as before, in PBS (not mounted). Images were acquired on a Nikon Eclipse Ti-2 inverted microscope with a PlanFluor 4x/0.2NA objective and a Hamamatsu ORCA-Flash 4.0 V3 sCMOS monochrome camera (pixel resolution = 1.63 μm/px). DAPI was excited with a 395/25 nm LED (Lumencor, Spectra-X) and imaged using a 447/60 nm emission filter. PCL fibers were imaged using transmitted light. For the images in Fig 5A, cells were also stained with 1:400 rhodamine-phalloidin (Cytoskeleton) at the same time as the DAPI staining. Rhodamine-phalloidin was excited with a 550/15 nm LED and imaged using a 641/74 nm emission filter.

Spheroid size was measured manually from the initial images using the ellipse drawing tool in FIJI/ImageJ, which yielded major and minor axis measurements. Spheroids were roughly circular with a median aspect ratio (minor/major axis) of 0.91; therefore spheroid radii were approximated by averaging the two axis measurements and dividing by two. For the final images, cells were segmented from the DAPI fluorescence channel with a semi-automated custom Matlab script using a modification of the “Water” algorithm [41, 45]. The analyst chose a threshold, segmentation parameters, as well as exclusion criteria (min/max size; minimum circularity) to achieve an accurate segmentation. After segmentation, the distance between each cell and the initial spheroid perimeter was calculated. Cells that were closer to the center of the spheroid than the initial spheroid radius (i.e. that were within the perimeter) were excluded. Fig 5B plots the median cell migration distance per spheroid. Statistical differences were assessed using the Wilcox Rank Sum test. P-values were adjusted for multiple comparisons using the Benjamini-Hochberg (false discovery rate) method.

For migration persistence experiments (Fig 5C), HT-1080 cells were labeled with DiI prior to incorporation into spheroids. Spheroids were seeded as described above. After spheroids had attached, PCL substrates were inverted into glass-bottomed dishes (MatTek or Matsunami; No. 1.5 coverslip) with CCM1 and held against the glass bottom by weighing down with a PDMS ring (see above). This procedure was necessary because the PCL fibers were spun onto plastic coverslips and therefore needed to be imaged from the fiber side of the substrate. There was a gap between the cells/fibers and the glass surface; the cells were not in contact with the dish bottom. Timelapse imaging was performed on a Nikon Eclipse Ti-2 inverted microscope with a PlanFluor 10x/0.3NA objective and a Hamamatsu ORCA-Flash 4.0 V3 sCMOS monochrome camera (pixel resolution = 0.65 μm/px). Samples were maintained at 37°C with a Tokai-Hit stage-top incubation chamber. DiI fluorescence was excited with a 550/15 nm LED (Lumencor, Spectra-X) and emission was collected with a 641/74 nm emission filter. Persistence analysis was performed as above for Fig 4. All tracks were truncated to 6.5 h and those tracks with shorter durations were excluded from the analysis. Statistical comparisons in Fig 5C were performed using two-tailed t-Tests with Welch’s correction for unequal variances. P-values were adjusted for multiple comparisons using the Benjamini-Hochberg (false discovery rate) method.

### Analysis software and scripts

Image analysis was performed in the FIJI distribution of ImageJ (v. 1.53c) [46] and in MATLAB (Mathworks; R2019a update 3). Specific plugins used are listed in the relevant sections, above. Data analysis, statistical tests, and plotting were performed in R (v. 3.6.0) using RStudio (v. 1.2.1335) and the Tidyverse packages (v. 1.3.1). Analysis code and data are publicly available through the Open Science Framework at this DOI: 10.17605/OSF.IO/KQWDX.
